# Recurrent aneurysmatic bleeding of pancreaticoduodenal aneurysm due to median arcuate ligament syndrome: a case report

**DOI:** 10.1093/jscr/rjae364

**Published:** 2024-05-30

**Authors:** Kyra Hofmann, Anna Lareida, Thomas Bächler, Stefan Breitenstein, Patryk Kambakamba

**Affiliations:** Department of General Surgery, Kantonsspital Schaffhausen, 8208 Schaffhausen, Switzerland; Department of Surgery, Clinic for Visceral and Thoracic Surgery, Kantonsspital Winterthur, 8400 Winterthur, Switzerland; Department of Surgery, Clinic for Visceral and Thoracic Surgery, Kantonsspital Winterthur, 8400 Winterthur, Switzerland; Department of Surgery, Clinic for Visceral and Thoracic Surgery, Kantonsspital Winterthur, 8400 Winterthur, Switzerland; Department of Surgery, Clinic for Visceral and Thoracic Surgery, Kantonsspital Winterthur, 8400 Winterthur, Switzerland

**Keywords:** median arcuate ligament syndrome, coeliac trunc compression, MALS, pancreaticoduodenal artery aneurysm, aneurysmatic bleeding, Dunbar syndrome

## Abstract

Median arcuate ligament syndrome (MALS) involves coeliac artery compression, causing a range of symptoms from chronic pain to life-threatening complications. This case features a 52-year-old patient with recurrent retroperitoneal bleeding from MALS-related inferior pancreaticoduodenal artery aneurysms (PDAAs). Emergency interventions, including surgical bleeding control, angioplasty, percutaneous drainage, and median arcuate ligament release, were conducted. The case highlights challenges in diagnosing and managing MALS-related PDAA, emphasizing the importance of early identification and tailored interventions based on clinical symptoms and imaging. Surgical intervention to release the ligament is the primary treatment, with considerations for prophylactic intervention in PDAA cases. Lack of established PDAA management protocols underscores the need for prompt intervention to prevent complications. In conclusion, this report stresses the association between MALS and PDAA, advocating for early identification and tailored management to mitigate complications.

## Introduction

Median arcuate ligament syndrome (MALS), also known as Dunbar syndrome, is a rare condition resulting from extraluminal compression of the coeliac artery by the median arcuate ligament, a fibrous arch connecting the diaphragm’s crura. Typically affecting females aged 20 to 50, it presents with postprandial epigastric pain, weight loss, and an epigastric bruit. MALS may also cause exercise-induced pain and gastroparesis causing nausea and vomiting [1–3]. While MALS is often asymptomatic and considered a benign anatomical occurrence, it can lead to life threatening complications and has been linked to true-aneurysm formation in the pancreaticoduodenal arteries [3–8]. Pancreaticoduodenal artery aneurysms (PDAAs) constituting <2% of visceral artery aneurysms, often lead to severe complications if untreated. Their presence has been associated with celiac artery or superior mesenteric artery (SMA) stenosis [5–7]. This case report details a case presenting with sudden epigastric pain due to spontaneous active bleeding complicating MALS, underscoring the potential severity of the syndrome and its associated complications.

## Case report

**Figure 1:**
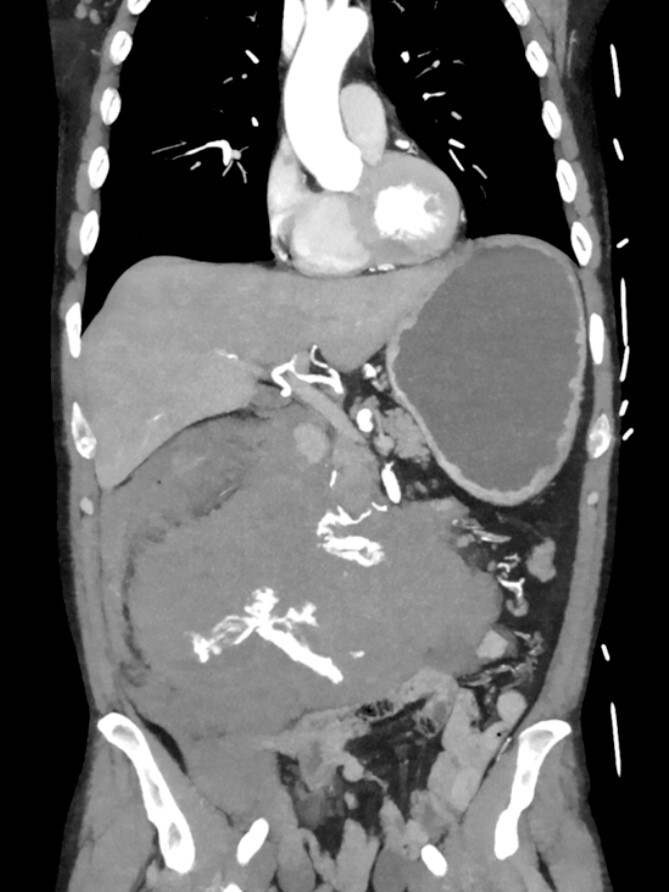
CTA showing an active bleeding likely from the pancreaticocuodenal artery with venous pooling and a large retroperitoneal hematoma.

A 52-year-old otherwise healthy patient was admitted to the emergency room of our hospital with an acute abdomen and hemodynamic instability. The computed tomography (CT) angiography revealed an active, most likely aneurysmal bleeding of the pancreaticoduodenal artery with venous pooling, along with a large retroperitoneal hematoma measuring ~2000 ml in volume (Fig. 1). Due to hemodynamic instability and the unavailability of interventionalists, the decision for emergent exploratory laparotomy was made. Intraoperatively was a ballooned and hemorrhagic retroperitoneum with incipient perforation of the mesenteric root observed. To gain exposure, an extended Kocher maneuver according to Cattell–Braasch was performed, necessitating a rightsided hemicolectomy. The bleeding was managed by ligating the pancreaticoduodenal artery. An Easy-Flow drainage was placed, and a split stoma was created. Intraoperatively, due to an estimated blood loss of over 4 L, transfusion therapy was administered following a massive transfusion protocol, along with extensive coagulation correction.

**Figure 2:**
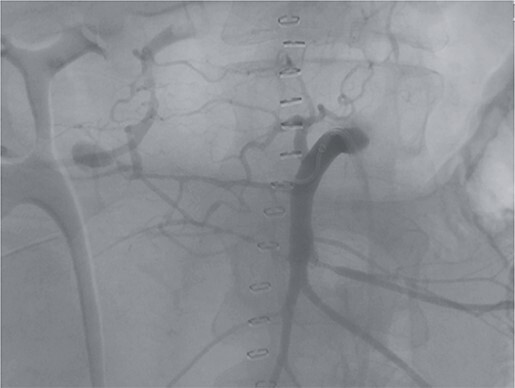
Angiography revealing the aneurysm of the pancreaticoduodenal arcade.

**Figure 3:**
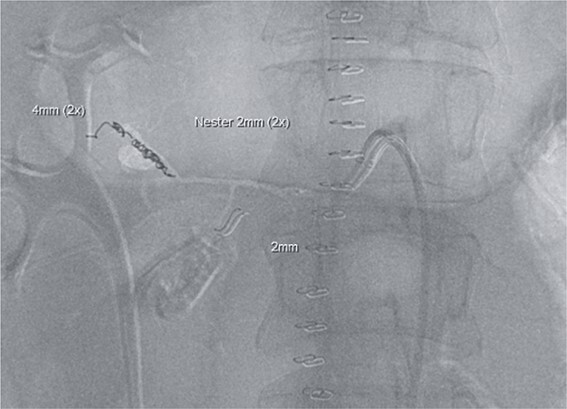
Postinterventional angiography, showing the aneurysm of the pancreaticoduodenal arcade, after interventional coiling.

Postoperatively, the patient achieved hemodynamic stabilization in the intensive care unit. On the sixth postoperative day, there was a recurrence of circulatory collapse in addition to relevant bloody discharge through the drains. CT imaging once again revealed an active bleeding of the pancreaticoduodenal artery, which was successfully treated interventionally using coiling (Figs 2 and 3).

**Figure 4:**
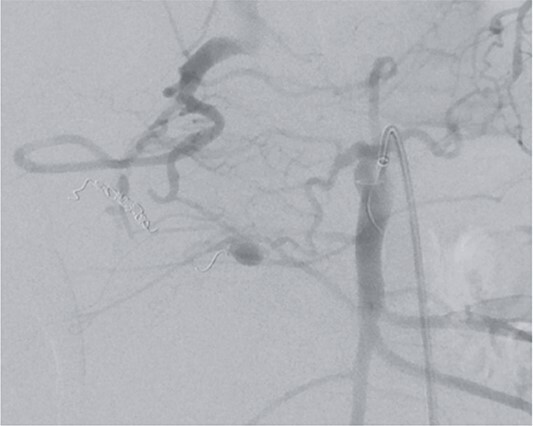
Angiography showing another aneurysm with active bleeding, proximally to the previously coiled site.

**Figure 5:**
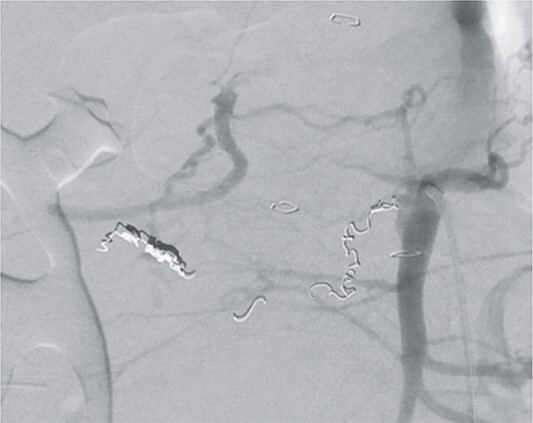
Postinterventional angiography, after interventional bleeding control, using coiling.

However, on the 16th day following the initial surgery, there was another active bleeding from the pancreaticoduodenal artery, distal to the previously placed coil. This bleeding was also managed interventionally (Figs 4 and 5).

**Figure 6:**
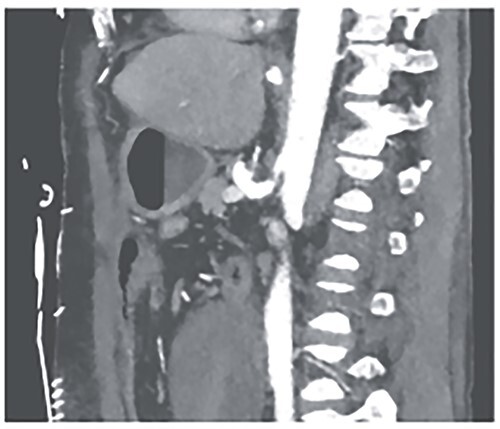
CTA showing a relevant stenosis of the celiac trunk.

**Figure 7:**
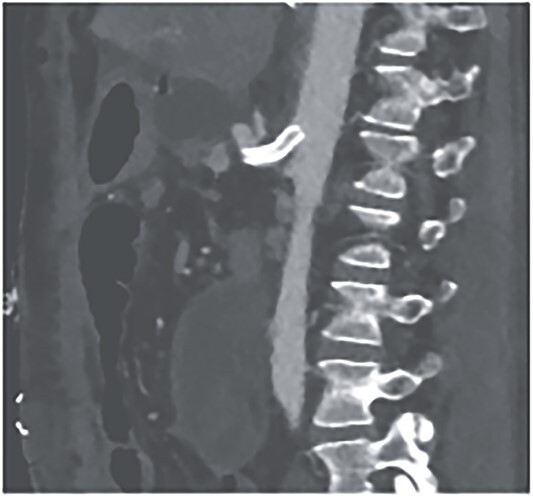
CTA showing successful intravascular sentient of celiac trunk.

**Figure 8:**
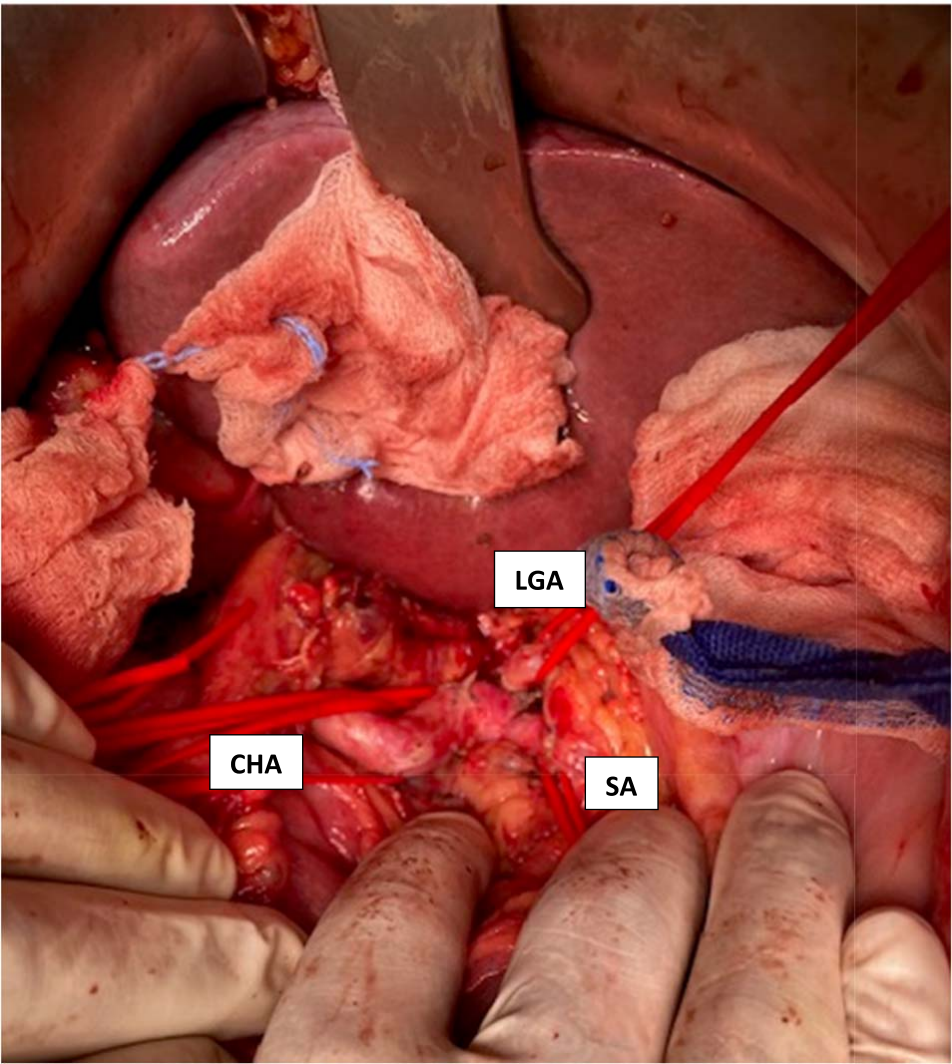
Intraoperative visualization of the coeliac trunk and its branches before decompression of the arcuate ligament (LGA = left gasric artery, CHA = common hepatic artery, SA = splenic artery).

We attributed the recurring aneurysmal bleedings of the pancreaticoduodenal arcade to extensive collateral circulation via the SMA, given an existing stenosis of the celiac trunk, most likely within the framework of a MALS (Fig. 6). To address this cause, a relaparotomy was performed the following day, decompressing the celiac trunk by splitting the median arcuate ligament (Fig. 8). Postoperatively, percutaneous transluminal angioplasty (PTA) with stenting of the celiac trunk was conducted for flow assurance (Fig. 7).

The further course was complicated by gastroparesis, likely caused by extraluminal compression of the duodenum by a persistent large retroperitoneal hematoma. Percutaneous drainage achieved successful evacuation of the collection and oral intake gradually improved thereafter.

There were no further bleeding episodes and the patient was discharged to rehabilitation under antibiotic therapy with the drainage *in situ*.

## Discussion

This case report brings attention to the complexities of MALS and its association with PDAAs, underscoring the challenges in diagnosis and management. MALS, a rare condition affecting the coeliac artery, triggers symptoms due to its compression by the median arcuate ligament. These symptoms vary widely, from asymptomatic presentation to chronic abdominal pain or possible complications like aneurysm formation and retroperitoneal hemorrhage [1–3].

Diagnosing MALS involves a combination of clinical symptoms, imaging studies, and Doppler ultrasound. The characteristic hooked appearance of the celiac artery during expiration on computed tomography angiography (CTA) aids in identification. However, the diagnosis can be elusive, especially in acute scenarios [1, 3, 5, 8].

Treating MALS primarily involves surgical intervention to release the ligament and improve blood flow. Endovascular techniques are less favored due to higher failure rates [8, 9]. PDAA, a rare possible complication of MALS, poses significant challenges in treatment, with considerations for intervention even in asymptomatic cases to prevent rupture [8, 9].

Aneurysms occurring in the inferior pancreaticoduodenal artery are typically due to conditions like atherosclerosis, pancreatitis, mycotic or bacterial infections, or trauma. There are also numerous case reports that describe an association between MALS and PDAAs, with no causal mechanism yet proven for this relationship. The association may be coincidental, as asymptomatic stenoses of the celiac trunk are highly common. Previous research has suggested that coeliac trunk narrowing leads to alterations in blood flow dynamics, which could cause increased blood circulation in the collateral arteries from the SMA to the coeliac artery leading to aneurysm formation [3–9].

There are currently no established treatment protocols for managing pancreaticoduodenal aneurysms. Consensus among experts suggests that prompt treatment upon detection is advisable, yet early identification to prevent rupture is rare. Increased arterial wall shear stress has been implicated in the development, enlargement, and rupture of these aneurysms. Unlike other types of aneurysms, the risk of rupture is not directly related to the diameter of the aneurysm itself [7–9]. Approximately 7–15% of PDAAs are associated with gastrointestinal hemorrhage, predominantly within the retroperitoneal space. When rupture occurs, mortality rates can reach up to 50%. Early treatment of true pancreaticoduodenal aneurysms is critical to prevent life threatening complications, due to a high risk of spontaneous rupture [7].

This report highlights the complex connection between MALS and PDAA, stressing the importance of early detection and tailored treatment approaches. To gain a deeper understanding of the link between MALS and PDAA development, it is crucial to systematically screen MALS patients for aneurysms.
